# Letter to the editor concerning ‘Association between sarcopenia index and the risk of second hip fracture in older adults’

**DOI:** 10.1016/j.jnha.2025.100601

**Published:** 2025-06-10

**Authors:** Li Liu

**Affiliations:** Jining Medical University, Jining, 272002, Shandong, China

Dear Editor,

Recently, we read and discussed an article titled "Association between sarcopenia index and the risk of second hip fracture in older adults" published by Yu et al. [1]. This study mainly evaluated the relationship between the sarcopenia index (SI) and the risk of second hip fracture. The study found that even after fully adjusting for the influence of potential modifiers such as age, sex, and clinical factors, SI still had high predictive accuracy and was an important predictor of second hip fracture in older adults. We greatly appreciate the authors' contributions and have several questions to discuss with them.

First, postoperative nutritional supplementation has been shown to help prevent complications in elderly patients after hip fracture [2]. Low vitamin D levels are also associated with an increased risk of hip fracture in elderly patients [3]. Therefore, postoperative nutritional supplementation and vitamin D intake may reduce the risk of second hip fracture. However, this study did not describe the postoperative follow-up of patients and lacked data on the nutritional status and serum vitamin D levels of patients who did not experience a second hip fracture, which may affect the accuracy of the study results.

Second, a study [4] has shown that a delay in community rehabilitation exercises for 2 weeks after discharge increases the risk of recurrent hip fracture by 15% in hip fracture patients, so timely postoperative rehabilitation exercises are crucial for preventing recurrent fractures. However, this study did not mention the rehabilitation exercise status of patients after discharge, which may affect the results.

In addition, a study by Wang et al. [5] showed that femoral neck areal bone mineral density (aBMD) is an important predictor of second hip fracture within the first year, while hip muscle density is an important predictor of second hip fracture after the second year. However, this study did not pay attention to the time interval between the two hip fractures and lacked data on femoral neck aBMD, making it impossible to exclude the potential influence of femoral neck aBMD, which may bias the results.

Finally, we once again thank the authors for their contributions to this study and look forward to their discussion on the above issues.

## Consent for publication

All authors gave their consent for publication.

## Ethics approval and consent to participate

Not applicable.

## Funding

There is no funding.

## Availability of data and materials

Not applicable.

## Declaration of competing interest

The authors declare that they have no known competing financial interests or personal relationships that could have appeared to influence the work reported in this paper.
